# Comparing psychotropic medication prescribing in personality disorder between general mental health and psychological services: retrospective cohort study

**DOI:** 10.1192/bjo.2021.34

**Published:** 2021-03-25

**Authors:** Giouliana Kadra-Scalzo, Jacqueline Garland, Stephen Miller, Chin-Kuo Chang, Marcella Fok, Richard D. Hayes, Paul Moran, Hitesh Shetty, Allan H. Young, Robert Stewart

**Affiliations:** Institute of Psychiatry, Psychology and Neuroscience, King's College London, UK; Croydon Personality Disorder Service, South London and Maudsley NHS Foundation Trust, UK; Croydon Personality Disorder Service, South London and Maudsley NHS Foundation Trust, UK; Institute of Psychiatry, Psychology and Neuroscience, King's College London, UK; Global Health Program, College of Public Health, National Taiwan University, Taiwan; and Institute of Epidemiology and Preventive Medicine, College of Public Health, National Taiwan University, Taiwan; Institute of Psychiatry, Psychology and Neuroscience, King's College London, UK; and Waterview Centre, Central and North West London NHS Foundation Trust, UK; Institute of Psychiatry, Psychology and Neuroscience, King's College London, UK; Centre for Academic Mental Health, Department of Population Health Sciences, Bristol Medical School, University of Bristol, UK; BRC Nucleus, South London and Maudsley NHS Foundation Trust, UK; Institute of Psychiatry, Psychology and Neuroscience, King's College London, UK; Institute of Psychiatry, Psychology and Neuroscience, King's College London, UK; and BRC Nucleus, South London and Maudsley NHS Foundation Trust, UK

**Keywords:** Personality disorder, psychological services, general mental health, medication prescribing, psychotropics

## Abstract

**Background:**

Although no drugs are licensed for the treatment of personality disorder, pharmacological treatment in clinical practice remains common.

**Aims:**

This study aimed to estimate the prevalence of psychotropic drug use and associations with psychological service use among people with personality disorder.

**Method:**

Using data from a large, anonymised mental healthcare database, we identified all adult patients with a diagnosis of personality disorder and ascertained psychotropic medication use between 1 August 2015 and 1 February 2016. Multivariable logistic regression models were constructed, adjusting for sociodemographic, clinical and service use factors, to examine the association between psychological services use and psychotropic medication prescribing.

**Results:**

Of 3366 identified patients, 2029 (60.3%) were prescribed some form of psychotropic medication. Patients using psychological services were significantly less likely to be prescribed psychotropic medication (adjusted odds ratio 0.48, 95% CI 0.39–0.59, *P*<0.001) such as antipsychotics, benzodiazepines and antidepressants. This effect was maintained following several sensitivity analyses. We found no difference in the risk for mood stabiliser (adjusted odds ratio 0.79, 95% CI 0.57–1.10, *P* = 0.169) and multi-class psychotropic use (adjusted odds ratio 0.80, 95% CI 0.60–1.07, *P* = 0.133) between patients who did and did not use psychological services.

**Conclusions:**

Psychotropic medication prescribing is common in patients with personality disorder, but significantly less likely in those who have used psychological services. This does not appear to be explained by differences in demographic, clinical and service use characteristics. There is a need to develop clear prescribing guidelines and conduct research in clinical settings to examine medication effectiveness for this population.

At present, drugs are not licensed specifically for the treatment of personality disorder.^1,2^ However, pharmacological treatment in clinical practice remains common.^3^ Indeed, the majority of people with a personality disorder who attend secondary mental health services are prescribed psychotropic medication, often over extended time periods.^4^ This is of concern for a number of reasons. Evidence suggests that psychotropic medication prescribing for this population is frequently outside licensed indications, and the recommended regular monitoring is not being done. Furthermore, multi-class psychotropic use has also been found to be common in this population.^3–5^ This is especially important given that polypharmacy is associated with physical health problems, such as weight gain, diabetes, metabolic syndrome, dyslipidaemia and prolonged QT and PR intervals,^6–8^ and detrimental health outcomes, such as hospital readmissions and mortality.^9,10^ Although there is little research into the factors influencing prescribing in this population, there is some evidence to suggest that patients with a personality disorder attending specialist services (including specialist personality disorder and psychological treatment) are less likely to be prescribed medication than those in receipt of general psychiatric care.^3^ However, existing research has examined small samples^3,4^ and focused predominately on more common subtypes of personality disorder, such as emotionally unstable subtype.^11,12^ The existence of an association between the receipt of prescribed psychotropic medication and attendance for psychological treatment among patients with personality disorder has not been previously investigated. It might be anticipated that specialist psychological treatment services may be more likely to adhere to national guidelines recommending that psychotropics are not routinely prescribed to people with personality disorder. Yet, to date, this has not been formally tested. The aim of this study was to start addressing this gap by examining psychotropic medication use among adults with a personality disorder who receive secondary mental healthcare, and to investigate whether this is reduced in patients seen by psychological treatment services.

## Method

We carried out a retrospective cohort study, using South London and Maudsley NHS Foundation Trust (SLAM) secondary mental healthcare electronic records, accessed via the Clinical Record Interactive Search (CRIS). SLAM provides near-monopoly public mental health services for approximately 1.36 million residents across four London boroughs (Lambeth, Southwark, Lewisham and Croydon). CRIS was developed in 2008, and accesses de-identified electronic health records information for over 400 000 people in SLAM. The CRIS resource has been previously described in detail,^13,14^ and is approved by the Oxford Research Ethics Committee C (reference 08/H606/71 + 5) as a database for secondary analysis.

Using CRIS, we ascertained all patients aged ≥18 years who were in contact with SLAM mental health services between 1 August 2015 and 1 February 2016, and had received a diagnosis of a personality disorder (ICD-10^15^ codes F60–F69) before the end of the observation period (1 August 2015 and 1 February 2016). For each patient, we determined the most recent personality disorder diagnosis before the end of the observation window, also referred to as index personality disorder diagnosis. We also determined whether patients had attended an Improving Access to Psychological Therapies service or secondary care specialist psychotherapy service, in the observation window.

The primary outcome was psychotropic medication use in the observation window. We considered all antipsychotic, benzodiazepine, antidepressant and mood stabiliser medications listed by the 65th edition of the British National Formulary.^16^ In instances where multiple medications from different categories were prescribed within a period of 6 months (but not necessarily simultaneously), this is referred to as multi-class psychotropic use. Psychotropic medication data were extracted from SLAM's pharmacy-dispensing database and from structured and free-text fields (using a natural language processing application) in the source health records accessed by CRIS. We have described the procedure for data extraction in detail in a separate publication.^17^

In addition, we investigated the sociodemographic, clinical and service use characteristics of all patients in our cohort. Age was calculated at the time of the index personality disorder diagnosis. The remaining sociodemographic factors were derived from the entry closest to 1 August 2015. Ethnic group categories were collapsed into ‘British’, ‘other White’, ‘Black Caribbean’, ‘Black African’, ‘Asian’ and ‘other’, because of small numbers in some cells. Clinical symptom presence/severity was estimated from the Health of the Nation Outcome Scales (HoNOS) completed closest to the index personality disorder diagnosis. HoNOS is a routinely administered clinical outcome instrument in British mental health services, and comprises 12 items designed to measure behaviour, impairment, symptoms and social functioning.^18^ Items are scored on a scale of 0 (no problem) to 4 (severe to very severe problem). Because of small cell sizes, subscale scores were collapsed into three categories: 0, ‘not a problem’; 1, ‘minor problem requiring no action’; and 2–4, ‘significant problem’. In addition, we determined the nature of any serious mental illness diagnosis (ICD-10 codes F20, F25 and F31) recorded closest to the index personality disorder diagnosis, and whether the patients had ever received a diagnosis of depression (ICD-10 codes F32 and F33), anxiety (ICD-10 codes F40–42), post-traumatic stress disorder (ICD-10 code F43), alcohol use disorder (ICD-10 code F10) or substance use disorder (ICD-10 codes F11, F12 and F14). The presence or not of any in-patient mental healthcare at any point during the observation window was further recorded.

### Statistical analysis

Stata version 13 for Windows was used for all statistical analyses. We estimated the proportion of patients who had psychological therapies contact in the observation window, and examined the distribution of the sociodemographic, clinical and service use characteristics across the cohort. We further examined the prevalence of psychotropic medication use in all patients with a personality disorder diagnosis. Multivariable logistic regression models were built to examine the association between being seen by psychological therapies and receiving a psychotropic medication/s. Models were adjusted for age, gender, ethnicity, comorbid serious mental illness diagnosis (ICD-10 codes F20, F25 and F31), in-patient stay during the observation window and HoNOS score.

Several sensitivity analyses were carried out. We first tested whether the timing of the HoNOS assessment had an effect on the association between psychological services contact and psychotropic prescription, by restricting the analyses to those with HoNOS scores within a year of the index personality disorder diagnosis. The principal reasoning for this was that we wanted to use the HoNOS score that was most reflective of the patient's functioning around the time of diagnosis. We further tested whether or not having a defined subtype of personality disorder had an effect on the association. In addition, we restricted the analysis to patients with an index personality disorder diagnosis within the past 2 years, to test whether this had an effect on the association. To address potential confounding by indication, we used a standard propensity score method, where the propensity score was the probability of receiving psychological service contact based on a model with all variables described above. The propensity scores were then used to identify patients whose scores indicated they have the potential to be seen or not seen by psychological therapies services in the observation period. In other words, those with extreme propensity scores (i.e. would always be likely to be seen by psychological services or never likely to be seen by psychological services) were excluded. We then constructed a fully adjusted logistic regression model, and restricted the analysis to patients with this restricted range of propensity scores. Finally, we conducted multivariable logistic regression analyses to examine the association between psychological services use and the prescription of multiple categories of psychotropic medications.

## Results

We identified 3366 adults with a personality disorder who were receiving SLAM care between 1 August 2015 and 1 February 2016. Of these, 1057 (31.4%) did not have a specific personality disorder subtype indicated in their index diagnosis. Of the patients who had a subtype indicated, the most common were emotionally unstable (*n* = 1705, 50.7%) and dissocial (*n* = 155, 4.6%).

Of the sample, 728 (21.6%) patients received either Improving Access to Psychological Therapies service (*n* = 245, 7.3%) or specialist services contact (*n* = 548, 16.3%). Table 1 summarises the characteristics of the total cohort and by psychological services contact in the observation window. Overall, patients seen by psychological services were slightly younger, female, had a higher proportion of depression and anxiety, and more significant problems with non-accidental self-injury. Patients not seen by a psychological service had a higher proportion of ever receiving a comorbid diagnosis of serious mental illness, alcohol and/or substance use, and higher in-patient stay during the observation window.

**Table 1:** Cohort characteristics of patients with personality disorder diagnosis

Variables	Total cohort, *n* (%)	Patients seen by psychological services, *n* (%)	Patients not seen by psychological services, *n* (%)
Total	3366	728 (21.6)	2638 (78.4)
Sociodemographic and socioeconomic factors			
Age, years			
18–25	614 (18.2)	148 (20.3)	466 (17.7)
26–35	881 (26.2)	238 (32.7)	643 (24.4)
36–45	848 (25.2)	162 (22.3)	686 (26.0)
46–55	680 (20.2)	134 (18.4)	546 (20.7)
≥56	343 (10.2)	46 (6.3)	297 (11.3)
Gender			
Female	1942 (57.7)	538 (73.9)	1404 (53.2)
Male	1423 (42.3)	190 (26.1)	1233 (46.8)
Ethnicity group			
British	1919 (57.7)	405 (56.3)	1514 (58.0)
White other	283 (8.5)	78 (10.9)	205 (7.9)
Asian	115 (3.5)	21 (2.9)	94 (3.6)
Black Caribbean	170 (5.1)	27 (3.8)	143 (5.5)
Black African	459 (13.8)	69 (9.6)	390 (14.9)
Other	382 (11.5)	119 (16.5)	263 (10.1)
Clinical factors			
Comorbid diagnosis			
Serious mental illness (ICD-10 codes F20, F25, F31) (closest to personality disorder diagnosis)	1563 (46.4)	181 (24.9)	1382 (52.4)
Depression (ICD-10 codes F32 and F33), ever	1441 (42.8)	380 (52.2)	1061 (40.2)
Anxiety (ICD-10 codes F40–F42), ever	596 (17.7)	169 (23.2)	427 (16.2)
Post-traumatic stress disorder (ICD-10 code F43), ever	635 (18.9)	152 (20.9)	483 (18.3)
Alcohol (ICD 10: F10), ever	565 (16.8)	80 (10.9)	485 (18.4)
Substance use (ICD-10 codes F11, F12, F14), ever	451 (13.4)	38 (5.2)	413 (15.7)
HoNOS scale items			
Overactive and aggressive behaviour			
Not a problem	1289 (43.0)	299 (42.3)	990 (43.2)
Minor problem	803 (26.8)	206 (29.1)	597 (26.1)
Significant problem	905 (30.2)	202 (28.6)	703 (30.7)
Non-accidental self-injury			
Not a problem	1886 (63.1)	367 (52.1)	1519 (66.5)
Minor problem	473 (15.8)	148 (20.9)	325 (14.2)
Significant problem	631 (21.1)	190 (27.0)	441 (19.3)
Problems with drinking or drugs			
Not a problem	1792 (60.0)	445 (63.3)	1347 (59.0)
Minor problem	363 (12.2)	95 (13.5)	268 (11.7)
Significant problem	831 (27.8)	163 (23.2)	668 (29.3)
Cognitive problems			
Not a problem	2032 (67.8)	559 (79.1)	1473 (64.4)
Minor problem	550 (18.4)	87 (12.3)	463 (20.2)
Significant problem	413 (13.8)	61 (8.6)	352 (15.4)
Physical illness or disability			
Not a problem	1663 (55.6)	442 (62.6)	1221 (53.4)
Minor problem	522 (17.4)	102 (14.4)	420 (18.4)
Significant problem	806 (26.9)	162 (22.9)	644 (28.2)
Hallucinations and delusions			
Not a problem	1889 (63.2)	576 (81.6)	1313 (57.5)
Minor problem	431 (14.4)	68 (9.6)	363 (15.9)
Significant problem	669 (22.4)	62 (8.8)	607 (26.6)
Problems with depressed mood			
Not a problem	602 (20.1)	72 (10.2)	530 (23.2)
Minor problem	764 (25.5)	107 (15.1)	657 (28.7)
Significant problem	1629 (54.4)	528 (74.7)	1101 (48.1)
Problems with relationships			
Not a problem	585 (19.6)	113 (15.9)	472 (20.7)
Minor problem	672 (22.5)	109 (15.4)	563 (24.7)
Significant problem	1730 (57.9)	485 (68.6)	1245 (54.6)
Problems with activities of daily living			
Not a problem	1391 (46.6)	376 (53.2)	1015 (44.5)
Minor problem	680 (22.8)	158 (22.4)	522 (22.9)
Significant problem	915 (30.6)	173 (24.5)	742 (32.6)
Problems with living conditions			
Not a problem	1751 (59.7)	467 (66.9)	1284 (57.5)
Minor problem	536 (18.3)	102 (14.6)	434 (19.4)
Significant problem	645 (22.0)	129 (18.5)	516 (23.1)
Problems with work			
Not a problem	1232 (41.7)	326 (46.2)	906 (40.3)
Minor problem	707 (23.9)	165 (23.4)	542 (24.1)
Significant problem	1015 (34.4)	215 (30.4)	800 (35.6)
Service use			
In-patient stay during window (binary)	432 (12.8)	56 (7.7)	376 (14.2)

HoNOS, Health of the Nation Outcome Scales.

In total, 2029 (60.3%) patients were taking some form of psychotropic medication. Of these, 997 (49.1%) were prescribed only one psychotropic and 1032 (50.9%) had multi-class psychotropic use. Table 2 describes the prevalence of specific psychotropic medication categories across the cohort. Antipsychotics (38.8%) and antidepressants (33.9%) were the most commonly prescribed medications.

**Table 2:** Psychotropic medication use among patients diagnosed with personality disorder

Class of psychotropic used^a^	Total cohort (*N* = 3366), *n* (%)	Patients seen by psychological services (*n* = 728), *n* (%)	Patients not seen by psychological services (*n* = 2638), *n* (%)
Antipsychotic	1306 (38.8)	123 (16.9)	1183 (44.8)
Benzodiazepine	634 (18.8)	86 (11.8)	548 (20.8)
Antidepressant	1143 (34.0)	241 (33.1)	902 (34.2)
Mood stabiliser	393 (11.7)	58 (8.0)	335 (12.7)
Multiple psychotropic use	1032 (50.9)	139 (43.7)	893 (52.2)

a. Individual groups are not mutually exclusive.

Table 3 summarises the multivariable logistic regression models for the association between receiving psychological therapies contact and being prescribed any psychotropic medication. In summary, patients in contact with psychological services were significantly less likely to be prescribed psychotropic medication. This association was sustained after adjusting for a number of sociodemographic, clinical and service use factors (odds ratio 0.48, 95% CI 0.39–0.59, *P*<0.001), and was further maintained following a propensity score-restricted (odds ratio 0.48, 95% CI 0.39–0.59, *P*<0.001) and adjusted analysis (odds ratio 0.47, 95% CI 0.39–0.56, *P*<0.001).

**Table 3:** Multivariable logistic regression analysis of the association between psychological services use and psychotropic medication prescribing

	Odds ratio (95% CI)	*P-*value
Unadjusted model (*N* = 3366)	0.42 (0.36–0.49)	<0.001
Fully adjusted model^a^ (*n* = 2864)	0.48 (0.39–0.59)	<0.001
Sensitivity analyses for fully adjusted model		
Restricted to sample with HoNOS obtained within a year of receiving a personality disorder diagnosis (*n* = 2505)	0.49 (0.39–0.61)	<0.001
Restricted to patients with undefined personality disorder (*n* = 889)	0.58 (0.36–0.93)	0.024
Restricted to patients with most recent personality disorder diagnosis within the past 2 years (*n* = 2106)	0.49 (0.39–0.62)	<0.001
Propensity score restricted and adjusted model (*n* = 2834)	0.48 (0.39–0.59)	<0.001

HoNOS, Health of the Nation Outcome Scales.

a. Model adjusted for age, gender, ethnicity, comorbid serious mental illness diagnosis (ICD-10 codes F20, F25 and F31), in-patient stay during the observation window and HoNOS score.

Table 4 summarises unadjusted and adjusted logistic regression models, which examined the association between having contact with psychological therapies services and using different types of psychotropic medication. In the fully adjusted models, individuals in contact with psychological services were significantly less likely to use antipsychotics (odds ratio 0.42, 95% CI 0.33–0.54, *P*<0.001), benzodiazepines (odds ratio 0.62, 95% CI 0.46–0.84, *P* = 0.002) and antidepressants (odds ratio 0.73, 95% CI 0.60–0.90, *P* = 0.003). These associations were maintained after several sensitivity analyses, but no association was found with mood stabiliser use. Lastly, we found no significant association after adjustment between psychological service contact and receiving medications from multiple psychotropic categories (odds ratio 0.80, 95% CI 0.60–1.07, *P* = 0.133) (Supplementary Table 1 available at https://doi.org/10.1192/bjo.2021.34).

**Table 4:** Multivariable logistic regression analysis of the association between psychological services use and different types of psychotropic medication prescribing (*N*=3366)

Psychotropic groups	Antipsychotics		Benzodiazepines		Antidepressants		Mood stabilisers	
	Odds ratio (95% CI)	*P*-value	Odds ratio (95% CI)	*P*-value	Odds ratio (95% CI)	*P*-value	Odds ratio (95% CI)	*P*-value
Unadjusted model	0.25 (0.20–0.31)	<0.001	0.51 (0.40–0.65)	<0.001	0.95 (0.80–1.13)	0.583	0.60 (0.44–0.79)	<0.001
Fully adjusted model^a^	0.42 (0.33–0.54)	<0.001	0.62 (0.46–0.84)	0.002	0.73 (0.60–0.90)	0.003	0.79 (0.57–1.10)	0.169
Sensitivity analyses								
Model^b^	0.41 (0.31–0.53)	<0.001	0.67 (0.49–0.91)	0.01	0.74 (0.60–0.92)	0.005	0.82 (0.58–1.17)	0.272
Model^c^	0.25 (0.13–0.49)	<0.001	0.71 (0.37–1.35)	0.293	1.04 (0.64–1.68)	0.879	0.81 (0.35–1.87)	0.625
Model^d^	0.46 (0.35–0.59)	<0.001	0.67 (0.48–0.91)	0.011	0.74 (0.59–0.92)	0.007	0.77 (0.54–1.10)	0.157

HoNOS, Health of the Nation Outcome Scales.

a. Model adjusted for age, gender, ethnicity, comorbid serious mental illness diagnosis (ICD-10 codes F20, F25 and F31), in-patient stay during the observation window and HoNOS score.

b. Fully adjusted model where HoNOS was obtained within a year of receiving a personality disorder diagnosis.

c. Fully adjusted model where analysis was restricted to patients who did not have a defined personality disorder category.

d. Fully adjusted model where analysis was restricted to patients who have had their most recent personality disorder diagnosis within the past 2 years.

## Discussion

In one of the largest observational studies of its kind, we examined recorded psychotropic medication prescribing in a cohort of patients diagnosed with personality disorder, and compared this between groups who did and did not use psychological services. Consistent with previous literature, the most commonly diagnosed personality disorder was the emotionally unstable type,^11,19,20^ the majority of the patients in the sample were receiving psychotropic medication from at least one class, just over a half were prescribed medications from two or more classes,^4,11^ and antipsychotics and antidepressants were the most commonly prescribed medications.^3,4,11^

Patients with personality disorder who were using psychological services were slightly younger, had less comorbid serious mental illness, more comorbid depression and anxiety, and less functional problems, such as activities of daily living impairment and problems with living conditions and work. They also had lower in-patient stay during the observation window, compared with patients not seen by psychological services. In agreement with Crawford et al,^3^ we found that patients who had contact with psychological services were less likely to be prescribed psychotropic medication in general, and more specifically, were less likely to receive antipsychotics, benzodiazepines and antidepressants. These associations remained largely unchanged after several sensitivity analyses. One possible explanation for these results could be that patients seen by psychological services are generally more stable, with fewer symptoms. However, we adjusted for severity of functional problems (as measured by HoNOS), and the presence of comorbid diagnoses, with little effect on the size of the associations. We also took account of confounding by indication, in other words the probability that medication prescription (or the reduction in this occurring) is a marker of other clinical features that are triggering referral to psychological services. To test for this possible explanation, we used a propensity score restriction and adjustment, and the results remained unchanged, suggesting that differences in the characteristics between the two groups could not explain why patients with psychological service contact were less likely to receive psychotropic medication. There is a need to understand factors that may influence psychotropic prescribing in this population. It is not known whether differences in psychotropic medication use reflect patient choice between medication and psychological treatment, or whether it is a function of service factors such as fewer prescribers in psychological treatment settings. Considering the observed differences from a service delivery perspective, it is not known whether medication is being withheld from a group of patients that could potentially benefit from it, or whether psychological treatment settings facilitate collaborative, judicious decision-making around medication use. Patient factors, such as lack of psychological mindedness and choosing not to engage in therapy, may preclude access to available psychological treatments,^21^ and medication may provide an alternative therapeutic approach in general psychiatry settings. Patients with personality disorder may wish to take psychotropic medication to obtain immediate relief from symptoms such as paranoia, affective dysregulation or anxiety, and may resist attempts to reduce or stop medication. It has also been suggested that the act of prescribing constitutes a way of relating to patients, which may be reflective of the psychopathology of the condition^22^ such that the act of prescribing itself may symbolise a tangible act of care. In the absence of psychological treatment, there may be an expectation to prescribe for the purposes of providing such care and attenuating distress.

Similar to previous findings,^3^ we found low mood stabiliser use in our cohort. Our results suggest that there was no difference in the likelihood of being prescribed mood stabilisers between the patients who were in contact with psychological services and those who were not. However, this lack of effect could have been a result of a lack of statistical power, given the infrequent use of mood stabilisers in the cohort overall. Given the teratogenicity of several mood stabilisers, avoidance of prescribing in women of childbearing age may account for the low use of mood stabilisers in the sample. Alternatively, the low level of mood stabiliser use could be because of the lack of consistent evidence to support their clinical efficacy for patients with personality disorder.^23^ Consistent with previous research, we found that a high proportion of the patients in our sample had multi-class psychotropic use^5,11^ ; however, we found no difference in the risk of multi-class psychotropic use between patients who were receiving psychological service and those who were not. Therefore, our findings do not support previously proposed hypotheses that patients seen by psychological service are less likely to be offered medication treatment as a result of receiving alternative treatments.^3^ An alternative hypothesis could be that patients who receive multi-class polypharmacy have either an inherently more severe personality disorder diagnosis or more severe comorbid mental disorders.

This study had several strengths. We examined a large and representative sample of patients seen by secondary mental health services. Although the sample was drawn from a single service provider, the heterogeneity of individual clinicians and teams are likely to outweigh any homogeneity imposed by the organisation, and our findings should be broadly reflective of real-world clinical practice in the UK.^14^ Furthermore, utilising information available from de-identified electronic health records allowed us to identify and adjust for rich and diverse contextual information, reducing residual confounding. We further limited the effect of confounding by indication, through applying a propensity score method as a sensitivity analysis. However, several potential limitations also need to be considered. SLAM, like London, has a substantially higher proportion of minority ethnic groups, and higher presentation of people in both the lowest and highest socioeconomic groups, compared with England,^14^ and this needs to be considered when drawing inferences. Although we adjusted for multiple confounders, it is possible that some residual confounding may have occurred. We were unable to measure factors such as duration of illness or stages of treatment as patients entered the observation period. Nor could we capture psychotropic prescribing reasons, which may have given information about possible mechanisms underlying our findings. Also, despite this being one of the largest observational studies in this filed, because of low numbers of certain personality disorder subtypes, we were unable to investigate medication use by personality disorder subtype. Nonetheless, our findings remain highly relevant as the field of personality disorder is moving toward the abandonment of subtypes of personality disorder and the retention of a single category of personality disorder.^24^ Lastly, clinical symptoms were measured at one point in time. Capturing clinical symptoms over a period and examining changes over time could have provided a better understanding of how symptoms are related to medication use.

This study has important clinical implications. Improving the quality of psychotropic medication use in people with personality disorder is a clinical priority. National Institute for Health and Care Excellence guidelines^1^ currently provide no guidance, although there is a clear need for clinicians to be better supported in their decision-making. Although American Psychiatric Association guidelines^2^ provide some support in relation to emotionally unstable personality disorder, evidence that most prescribing does not clearly target comorbid conditions^11^ is important in considering their clinical utility. Development of formal guidance on conducting medication reviews for patients with personality disorder is needed to ensure appropriate and rational prescribing.

We suggest that the broad framework of medication optimisation, which provides a person-centred approach to the safe and effective use of medication by using the best available evidence to guide shared decision-making about treatment,^24^ may support this aim. Medication review, a core part of a medication optimisation process, involving the structured and critical evaluation of prescribed medication, should be offered to all patients with personality disorder, including those in psychological treatment services.

There is also a need for clinical trials to ascertain the efficacy of psychotropic medication in this patient group. However, perhaps both more urgently and more practically, research in real-world clinical settings is needed to examine medication effectiveness. Efficacy studies with necessarily strict inclusion and exclusion criteria demand a degree of patient uniformity that is seldom seem in clinical practice, and the complexities of setting up efficacy studies mean that this is perhaps a longer-term goal. Research should not exclusively focus on emotionally unstable personality disorder given that almost half of the patients in our cohort had an undefined personality disorder or another subtype.

## Data Availability

The data that support the findings of this study are available on request from the corresponding author (G.K.-S.). The data are not publicly available due to the Information Governance framework and Research Ethics Committeeapproval in place concerning CRIS data use.

## Supplementary material

For supplementary material accompanying this paper visit http://dx.doi.org/10.1192/bjo.2021.34. click here to view supplementary material
