# Supplementary material for: Comparing psychotropic medication prescribing in personality disorder between general mental health and psychological services: retrospective cohort study
Source: BJPsych Open. 2021 Mar 25;7(2):e72. doi: 10.1192/bjo.2021.34 (PMC8058890; doi:10.1192/bjo.2021.34)
Supplement: Supplementary file 1 [file S205647242100034Xsup001.docx]

**Supplementary Table 1. Multivariable logistic regression analysis of the association between psychological services use and multi-class psychotropic^a^ medication use^b^ (n=3,366).**

|  | OR (95% CI) | p value |
| --- | --- | --- |
| Unadjusted Model | 0.71 (0.56- 0.91) | **P= 0.006** |
| Fully Adjusted Model^c^ | 0.80 (0.60- 1.07) | P=0.133 |
| Fully Adjusted Model^d^ | 0.79 (0.58- 1.06) | P= 0.116 |
| Fully Adjusted Model^e^ | 0.80 (0.59- 1.08) | P=0.151 |

^a^ Multiple class psychotropic medications were prescribed in a period of six months, however not necessarily simultaneously

^b^ The reference group is patients who have received one psychotropic medication

^c^ Model adjusted for age, gender, ethnicity, comorbid serious mental illness diagnosis include ICD 10: F20, F25, F31, inpatient stay during the observation window and Honos score

^d^ Fully adjusted model where Honos was obtained within a year of receiving a personality disorder diagnosis

^e^ Fully adjusted model where analysis was restricted to patients who have had their most recent personality disorder diagnosis within the last 2 years
